# The role of nasopharyngeal examination and biopsy in the diagnosis of malignant diseases^☆^

**DOI:** 10.1016/j.bjorl.2018.04.006

**Published:** 2018-05-15

**Authors:** Necmi Arslan, Arzu Tuzuner, Alper Koycu, Songul Dursun, Sema Hucumenoglu

**Affiliations:** a University of Health Sciences, Ankara Training and Research Hospital, Department of Otolaryngology, Head and Neck Surgery, Ankara, Turkey; b Baskent University Hospital, Department of Otolaryngology, Head and Neck Surgery, Ankara, Turkey; c University of Health Sciences, Ankara Training and Research Hospital, Department of Pathology, Ankara, Turkey

**Keywords:** Nasopharynx carcinoma, Nasopharynx biopsy, Endoscopic nasopharyngoscopy, Carcinoma de nasofaringe, Biópsia de nasofaringe, Nasofaringoscopia endoscópica

## Abstract

**Introduction:**

In direct proportion to the increasing rate of nasopharynx examinations applied, the early diagnosis and treatment of lesions in this region is possible. At times the clinical findings and the biopsy results are not consistent, so biopsies may have to be repeated.

**Objectives:**

The aim of this study was to evaluate the distribution of pathology test results obtained from cases of nasopharynx biopsy, to determine with which methods determination most often was made, and to investigate which kinds of cases required the biopsy to be repeated.

**Methods:**

The study included a total of 1074 patients (500 female, 574 male) who underwent nasopharyngeal biopsy in our clinic between June 2011 and June 2017. Data were obtained from patient records of age, gender, clinical findings, imaging findings if available and pathological diagnosis. The pathological diagnoses were separated into 3 main groups as chronic nasopharyngitis, benign cytology and malignant cytology.

**Results:**

The examinations resulted in 996 cases reported as chronic nasopharyngitis, 47 as benign cytology and 31 as malignant cytology. Of the 31 malignant lesions, diagnosis was made in 15 patients (48.4%) with a single biopsy, and in 16 patients (51.6%), as a result of the pathology report when 2 or more biopsies were taken. In the comparison of the benign and malignant lesions in respect of the need for repeated biopsies, the cases determined with malignancy were found to have a statistically significantly higher rate of repeated biopsy (*p* < 0.001).

**Conclusion:**

In comparison with cases of benign tumor, a statistically significantly greater number of repeated biopsies were required in cases diagnosed as malignant tumors to confirm the pathological diagnosis or when there was continued suspicion of malignancy. Therefore, when there is clinical suspicion, even if there are no findings of malignancy on the first biopsy, the biopsy should be repeated expeditiously.

## Resumo

**Introdução:**

Em proporção direta à taxa crescente de exames de nasofaringe que são feitos, o diagnóstico precoce e o tratamento de lesões nessa região têm sido possíveis. Nem sempre os achados clínicos e os resultados da primeira biópsia são consistentes, levando à necessidade de biópsias repetidas.

**Objetivos:**

Avaliar a distribuição dos resultados dos testes histopatológicos obtidos pela biópsia de nasofaringe, determinar quais métodos foram mais frequentemente usados na identificação e investigar os casos nos quais a biópsia precisou ser repetida.

**Método:**

O estudo incluiu 1.074 pacientes (500 mulheres, 574 homens) submetidos a biópsia de nasofaringe em nossa clínica entre junho de 2011 e junho de 2017. Os dados foram obtidos dos prontuários dos pacientes e incluíram idade, sexo, achados clínicos, achados de imagem e diagnóstico histopatológico. Os diagnósticos histopatológicos foram separados em três grupos principais como nasofaringite crônica, citologia benigna e citologia maligna.

**Resultados:**

Os exames resultaram em 996 casos laudados como nasofaringite crônica, 47 como citologia benigna e 31 como citologia maligna. Das 31 lesões malignas, o diagnóstico foi feito em 15 (48,4%) com uma única biópsia e em 16 (51,6%), quando duas ou mais biópsias foram realizadas. Na comparação das lesões benignas e malignas em relação à necessidade de biópsias repetidas, os casos determinados como sendo malignos mostraram uma taxa estatisticamente maior de biópsia repetida (*p* < 0,001).

**Conclusão:**

Em comparação com os casos de tumores benignos, um número estatisticamente maior de biópsias repetidas foi necessário em casos diagnosticados como tumores malignos, para confirmação do diagnóstico histopatológico ou na suspeita continuada de malignidade. Portanto, quando há suspeita clínica, mesmo que não haja achados de malignidade na primeira biópsia, ela deve ser repetida tão logo seja possível.

## Introduction

Several malignant and benign tumors are encountered in the nasopharynx, and even though findings such as otitis with effusion or a mass in the neck may be present, lesions may have an asymptomatic course until an advanced stage.^1^, ^2^, ^3^ Endoscopic nasopharyngoscopy applied in the ear, nose and throat examination can increase the rates of diagnosing nasopharynx cancer at an early stage.^4^, ^5^

The most frequently encountered findings in symptomatic cases are nasal obstruction, otitis media with effusion and epistaxis; endoscopic nasopharyngoscopy is the gold standard in clinical examination.^6^ In the presence of findings such as chronic middle ear effusion, a mass on the neck or a suspicious appearance determined on endoscopic nasopharyngeal examination, investigation of malignancy with a biopsy can be necessary, even if no specific focus is seen. Although many different scanning and specific examination methods have been described in the determination of nasopharyngeal malignancies, biopsy under endoscopy guidance remains indispensable for both a definitive diagnosis and pathological classification.^7^ When the clinical examination findings and the biopsy results are not consistent, it may be necessary to repeat the biopsy without any loss of time.

The aim of this study was to evaluate the distribution of pathology test results obtained from cases of nasopharynx biopsy, to determine with which methods determination most often was made, and to investigate which kinds of cases required repeat biopsy.

## Methods

This retrospective study included the records of 1074 patients (574 males, 500 females) who underwent endoscopic transnasal nasopharynx biopsy between June 2011 and June 2017 in the Ear, Nose and Throat (ENT) Clinic of Ankara Training and Research Hospital. Permission for the study was granted by the Education Planning Commission of Ankara Training and Research Hospital (Ref n° 0670/5618-04.01.2017). In the presence of findings such as chronic middle ear effusion, a mass in the neck or a suspicious appearance determined on endoscopic nasopharynx examination, transnasal endoscopic nasopharynx biopsy was performed. Patients in whom general anesthesia constituted a high risk and/or those who accepted local anesthesia underwent endoscopic transnasal nasopharynx biopsies under local anesthesia in the operating theatre. In other cases, the patients were operated on under general anesthesia. The patient records were examined and data were recorded including age, gender, clinical findings (nasal obstruction, otitis media with effusion, mass in the neck), imaging findings if available and pathological diagnosis. Immunohistochemical analysis was applied to all patients to define the tumor type. The pathological diagnoses were separated into 3 main groups: chronic nasopharyngitis, benign cytology and malignant cytology. In cases where the material was insufficient for a pathology report, those patients were excluded from the study. Two or more core and transitional deep biopsies were obtained from all patients. As the tumor localization was not fully specified in the records of all patients, no evaluation could be made of the nasopharyngeal tumor site.

Statistical analyses of the data were made using IBM SPSS for Windows Version 22.0 software. Numerical variables were stated as mean ± standard deviation (SD) and minimum–maximum values, and categorical variables as number (*n*) and percentage (%). Differences between the malignant and benign groups were examined with the Chi square test. A value of *p* < 0.05 was accepted as statistically significant.

## Results

A total of 904,609 patients were examined between June 2011 and June 2017 in the Ear, Nose and Throat (ENT) Clinic of Ankara Training and Research Hospital. Endoscopic transnasal nasopharynx biopsy was performed on 1074 patients (ratio of all examined patients: 0.118%). All patients underwent endoscopic transnasal nasopharynx biopsies under general or local anesthesia in the operating theatre and no major complications were recorded, other than minor bleeding that was controlled with bipolar cauterisation. The patients comprised 574 (53.4%) males and 500 (46.6%) females with a mean age of 43.2 ± 12.0 years (range 16–77 years). No difference was determined between the groups in respect of gender. The examinations resulted in 996 (92.7%) cases reported as chronic nasopharyngitis, 47 (4.4%) as benign cytology and 31 (2.9%) as malignant cytology. The numbers and percentages of the benign and malignant diagnoses are shown in Table 1.

**Table 1:** Demographic data.

	Number	Percentage
*Gender*		
Female	500	46.6
Male	574	53.4

*Result*		
Malignant	31	2.9
Benign	47	4.4
Chronic nasopharyngitis	996	92.7

*Result*		
Chronic nasopharyngitis	996	92.7
Inflammatory polyp	7	0.7
Lymphoid hyperplasia	30	2.8
Non-keratinizing undifferentiated carcinoma	19	1.8
Keratinizing undifferentiated carcinoma	2	0.2
Non-Hodgkin's lymphoma	3	0.3
Hodgkin's lymphoma	3	0.3
Anaplastic nasopharyngeal cancer	1	0.1
Angiofibroma	1	0.1
Granulomatous inflammation	4	0.4
Olfactory neuroblastoma	1	0.1
Malignant melanoma	1	0.1
Tonsil cancer	1	0.1
Papilloma	1	0.1
Thornwald cyst	4	0.4

	Mean ± SD	Min–Max
*Age (years)*	43.2 ± 12.0	16–77

The pathology reports of malignant diagnosis in 31 cases included non-keratinizing undifferentiated carcinoma in 19 (61.3%), anaplastic nasopharynx carcinoma in 1 (3.2%), keratinizing undifferentiated carcinoma in 2 (6.5%), non-Hodgkin's lymphoma in 3 (9.7%), Hodgkin's lymphoma in 3 (9.7%), olfactory neuroblastoma in 1 (3.2%), malignant melanoma metastasis in 1 (3.2%) and metastasis of tonsil carcinoma in 1 (3.2%) (Table 2).

**Table 2:** Distribution of malignant lesions.

	Number	Percentage
Non-keratinizing undifferentiated carcinoma	19	61.3
Keratinizing undifferentiated carcinoma	2	6.5
Non-Hodgkin's lymphoma	3	9.7
Hodgkin's lymphoma	3	9.7
Anaplastic nasopharyngeal cancer	1	3.2
Olfactory neuroblastoma	1	3.2
Malignant melanoma	1	3.2
Tonsil cancer	1	3.2

At the time of diagnosis of malignant lesions, computed tomography (CT) was requested in only 2 cases and in the remainder; the diagnosis was made as a result of existing symptoms and findings causing suspicion in the clinical examination. In the cases of benign lesions, a biopsy was taken after the lesion was determined on magnetic resonance imaging (MRI) in 8 cases and on CT in 4 (Table 3).

**Table 3:** Statistical analysis of the findings of the physical examination, number of biopsies and imaging methods used.

	Malignant lesions	Benign lesions	*p*
*Imaging*			
MRI	–	8	NA
CT	2	4	

*Findings*			
Effusion	2	–	NA
Mass in the neck	6	3	
Nasal obstruction	4	7	
Preseptal cellulitis	1	–	

*Number of biopsies*			
1	15 (48.4%)	46 (97.9%)	<0.001
>1	16 (51.6%)	1 (2.1%)	

If a mass in the neck conforming to malignancy criteria was discovered during the examination and neck imaging, or if persistent middle ear effusion with an ipsilateral or contralateral nasopharygeal mass was detected, biopsy was repeated without any loss of time. Of the 31 malignant lesions, diagnosis was made in 15 (48.4%) with a single biopsy, and in 16 (51.6%) the pathological diagnosis was reported as malignant when more than 1 biopsy was taken at different times. The diagnosis of malignant lesions in 16 cases with recurrent biopsy (2 or 3 separate biopsies) were non-keratinizing undifferentiated carcinoma in 12 (75%) cases, Hodgkin's lymphoma in 3 (18.75%), and non-Hodgkin's lymphoma in 1 (6.25%). Of the 5 patients from whom 3 separate biopsies at different times were taken, the definitive diagnosis was malignant in 3 cases as follows; non-keratinizing undifferentiated carcinoma, Hodgkin's lymphoma, and non-Hodgkin's lymphoma. Of the benign cases, diagnosis was made from a second biopsy in only 1 case. In the comparison of the benign and malignant lesions in respect of the need for repeated biopsies, the cases determined with malignancy were found to have a statistically significantly higher rate of repeated biopsy (*p* < 0.001) (Table 3).

## Discussion

Nasopharynx carcinoma has different characteristics from other head and neck epithelial tumors in respect of epidemiology, histopathology, findings, diagnosis and treatment modalities.^8^ As nasopharynx carcinoma is a tumor which grows silently and has a deep location extending from the region, it is an advanced stage tumor at the time of diagnosis of several diseases.^9^, ^10^ Several different scanning and specific examination methods have been described such as the serological examination of Epstein–Barr virus antibodies and smear sampling in addition to the use of MRI and CT as imaging methods. However, biopsy under endoscopic guidance remains indispensable both for definitive diagnosis and for pathological classification.^7^, ^8^, ^9^, ^10^

The sensitivity of endoscopic imaging in the diagnosis of nasopharynx carcinoma has been shown to be over 90%.^6^ In the current study, of the 1074 patients who underwent nasopharynx biopsy under endoscopy guidance, imaging methods were only applied to 14, as CT in 6 cases and MRI in 8. In the majority, diagnosis was made from biopsy when a suspicious lesion was observed on endoscopic nasopharyngoscopy or from blind nasopharynx biopsy when there was persistent unilateral chronic otitis media in the examination, or the presence of a malignant mass which was obviously not primary. However, in the literature there are reports that advocate the necessity for imaging before biopsy in cases of clinical suspicion.^6^, ^7^, ^8^, ^9^, ^10^, ^11^ According to some publications, imaging is necessary to be able to discount malignancy in cases of suspicious lesions such as those discovered with mild swelling or asymmetry determined in the endoscopic examination and thus the patient can be spared an unnecessary invasive intervention and a loss of workforce can be avoided.^11^ King et al.^6^ reported an accuracy rate of 95% for nasopharynx MRI in primary nasopharynx cancers.

In a study by Bercin et al.^11^ of 983 nasopharynx biopsies, 45 (4.6%) were reported as malignant. In the current study, the rate of malignancy was found to be 2.9%. Although the rate of malignancy in the current study was lower, the results are similar. The reason for the difference can be explained by the fact that the first preference of Bercin et al. for suspicious nasopharynx lesions (mild swelling or asymmetry) was to apply MRI examinations rather than biopsy and if contrast involvement, invasion of soft tissue or erasure of borders were determined on the MRI report, then a biopsy was performed.

According to Liu et al.,^12^ while malignant lymphomas are the second most common head and neck tumor, the most common location of their extra-nodal involvement is the Waldeyer lymphatic ring. In the current study, lymphoma (*n* = 6) was determined to be the second most common malignant tumor, which was consistent with literature.

Of the 31 malignant lesions in the current study, the pathological diagnosis of 16 (51.6%) could only be confirmed with repeated separate biopsies at different times. The diagnosis of malignant lesions of 16 cases with recurrent biopsy (2 or 3 separate biopsies) were non-keratinizing undifferentiated carcinoma in 12 (75%) cases, Hodgkin's lymphoma in 3 (18.75%), and non-Hodgkin's lymphoma in 1 (6.25%). A higher risk for recurrent biopsy was determined for non-keratinizing undifferentiated carcinoma. In the comparison of the benign and malignant lesions in respect to the need for repeated biopsies, the cases determined with malignancy were found to have a statistically significantly higher rate of repeated biopsy (*p* < 0.001). If the physician has suspicion of malignancy because of either the appearance of the lesion on endoscopic nasopharyngoscopy or other clinical findings, when the suspicion persists even if the results of the first biopsy are benign or there is chronic nasopharyngitis, then despite an increase in patient morbidity and cost-effectiveness, it can be considered appropriate to repeat the biopsy without any loss of time. It is clear that early diagnosis and treatment of the patient will increase survival rates.

Bercin et al.^11^ reported the sensitivity of MRI to be 88.2% in nasopharynx malignancies and the sensitivity of endoscopic biopsy to be 84.4%. This can be attributed to not applying imaging first in cases where problems could be experienced in determining the lesion location, especially in tumors with a deep location and that there could be negative biopsy results. The reason that there was a need for such a high rate of repeated biopsies to be able to reach a diagnosis of malignancy in the current study can be considered to be that the lesion location was not determined with preoperative imaging or that the biopsy was not taken from a sufficient depth.

A limitation of the current study was that preoperative imaging was not applied routinely to all patients with a suspicious lesion or to those who were being considered for biopsy. If there had been a sufficient number of preoperative images, these findings could have been compared with the endoscopic biopsy results. Under current conditions in Turkey, as there is a lengthy waiting time for an imaging appointment at our hospital, it is preferred to perform a biopsy in a short time to reduce the spread of the disease and provide diagnosis and treatment quickly, and, when suspicion of malignancy persists, to take repeated biopsies.

## Conclusion

In the vast majority of the patients in this study diagnosed with malignant and benign lesions, the determination of the lesion on the pre-diagnostic endoscopic nasopharyngoscopy image was seen to be the first finding without radiological examination and findings of disease outside the nasopharynx. Routine endoscopic examination of all ENT patients can be considered necessary.

In comparison with cases of benign tumor, a statistically significantly greater number of repeated biopsies were taken in cases diagnosed as malignant tumors to confirm the pathological diagnosis. Therefore, when there is clinical suspicion, even if there are no findings of malignancy on the first biopsy, it can be recommended that the biopsy should be repeated without any loss of time.

## Funding

Permission for the study was granted by the Education Planning Commission of Ankara Training and Research Hospital (Ref n° 0670/5618-04.01.2017).

## Conflicts of interest

The authors declare no conflicts of interest.
